# Selection and Validation of Reference Genes in Sudan Grass (*Sorghum sudanense* (Piper) Stapf) under Various Abiotic Stresses by qRT-PCR

**DOI:** 10.3390/genes15020210

**Published:** 2024-02-06

**Authors:** Fangyan Wang, Peng Li, Qiuxu Liu, Gang Nie, Yongqun Zhu, Xinquan Zhang

**Affiliations:** 1 College of Grassland Science and Technology, Sichuan Agricultural University, Chengdu 611130, China; wfy_zr@163.com (F.W.); lipeng_plant@163.com (P.L.); nieg17@sicau.edu.cn (G.N.); 2 Institute of Agricultural Resources and Environment, Sichuan Academy of Agricultural Sciences, Chengdu 610066, China; sicauliuqiuxu@163.com

**Keywords:** reference gene, Sudan grass, qRT-PCR, abiotic stress

## Abstract

Quantitative reverse transcription PCR (qRT-PCR) can screen applicable reference genes of species, and reference genes can be used to reduce experimental errors. Sudan grass (*Sorghum sudanense* (Piper) Stapf) is a high-yield, abiotic-tolerant annual high-quality forage with a wide range of uses. However, no studies have reported reference genes suitable for Sudan grass. Therefore, we found eight candidate reference genes, including *UBQ10*, *HIS3*, *UBQ9*, *Isoform0012931*, *PP2A*, *ACP2*, *eIF4α*, and *Actin*, under salt stress (NaCl), drought stress (DR), acid aluminum stress (AlCl_3_), and methyl jasmonate treatment (MeJA). By using geNorm, NormFinder, BestKeeper, and RefFinder, we ranked eight reference genes on the basis of their expression stabilities. The results indicated that the best reference gene was *PP2A* under all treatments. *eIF4α* can be used in CK, MeJA, NaCl, and DR. *HIS3* can serve as the best reference gene in AlCl_3_. Two target genes (*Isoform0007606* and *Isoform0002387*) belong to drought-stress-response genes, and they are highly expressed in Sudan grass according to transcriptome data. They were used to verify eight candidate reference genes under drought stress. The expression trends of the two most stable reference genes were similar, but the trend in expression for *Actin* showed a significant difference. The reference genes we screened provided valuable guidance for future research on Sudan grass.

## 1. Introduction

Sudan grass (*Sorghum sudanense* (Piper) Stapf) belongs to the sorghum family, native to Africa. It has many tillers, good palatability, and high nutritional value while suppressing weeds and increasing soil organic matter [1,2,3]. It is a favorite food for cattle, sheep, and fish. After consuming Sudan grass, fresh milk production can be improved, which can bring high economic benefits [4]. Therefore, Sudan grass is an important feed crop for the development of animal husbandry in many countries [5]. Marginal land is a kind of land with a poor climate, poor physical properties, and difficult to cultivate. It is widely distributed throughout the world. If marginal land can be effectively used, it can significantly fill the gap in the demand for high-quality forage grass [6]. However, abiotic stresses on marginal land, such as salinity, acid aluminum, and drought, threaten the production of Sudan grass [7,8,9]. Therefore, it is urgent to research the mechanism of resistance of Sudan grass to abiotic stress. To study gene expression in more detail and depth, it is important to pinpoint appropriate reference genes for Sudan grass.

Quantitative reverse transcription PCR (qRT-PCR) is often used in the field of molecular biology because of its strong specificity and high sensitivity [10]. In order to achieve precise test results, it is essential to employ suitable reference genes to normalize and standardize the expression levels of target genes [11]. At present, the ordinarily used evaluation method of reference genes is based on Ct values of qRT-PCR; widely used software includes geNorm ((ver.3.5), NormFinder (ver.0.953), and BestKeeper (ver.1.0) [12].

It is widely recognized that the optimal reference genes can vary depending on the species and specific situations [13]. *TUB-α* and *PGK* are optimal reference genes in *Toona ciliate*, but *PP2C59* and *UBC5B* are the most stable reference genes in leaves, and *HIS1* and *ACT7* can serve as reference genes in young stems under *Hypsipyla robusta* treatment [14]. *GADPH* is not optimal as a reference gene for ABA treatment in *Isodon rubescens,* while it is suitable to be used in the roots of *Avena sativa* L. under drought stress and the leaves under salt stress [15,16]. Under low-temperature pressure, the optimal reference genes in leaves of Caucasian clover (*Trifolium ambiguum* M. Bieb.) are *APA* and *EFTu-GTP*, and *RCD1* and *NLI2* are the most stable in the roots [17]. Meanwhile, different analysis methods will lead to different results due to their different algorithms [18]. *GAPC* and *TUB2* can serve as reference genes in various developmental stages of purple- and white-flower genotype (DPW) samples of *Allium wallichii* by GeNorm, *TUB2* and *EF1α* are optimal in DPW by NormFinder, and *UBC* is the best in DPW by BestKeeper [19]. Hence, identifying the most suitable reference genes among various plant varieties, growth stages, experimental treatments, and other conditions is important.

Usually, when studying gene expression, we will choose more than one gene to serve as reference genes for result correction to minimize experimental errors [20]. However, Web of Science (www.webofscience.com) did not include any information about the reference genes of Sudan grass. Only sorghum, which is in the same genus as Sudan grass, had reports on the reference genes. Therefore, we will fill the gap of the reference genes of Sudan grass through this experiment. In this study, eight reference genes were assessed through geNorm, NormFinder, BestKeeper, and RefFinder, namely, *Eukaryotic initiation factor 4 α* (*eIF4α*), *Histone H3* (*HIS3*), *Polyubiquitin 9* (*UBQ9*), *Polyubiquitin 10* (*UBQ10*), *Protein phosphatase 2A* (*PP2A*), *Actin gene* (*Actin*), *Acyl carrier protein 2* (*ACP2*), and *Isoform0012931*, in salt stress (NaCl), drought stress (DR), acid aluminum stress (AlCl_3_), and methyl jasmonate treatment (MeJA), to facilitate future research on Sudan grass.

## 2. Materials and Methods

### 2.1. Plant Material

Sudan grass cv. Chuansu No. 1, which was selected from the Sichuan Academy of Agricultural Sciences, was used in our research. Sudan grass seeds were sterilized by immersing them in 10% sodium hypochlorite solution for 5 min. Afterward, all of the Sudan grass seeds were rinsed three times with sterile distilled water to ensure thorough cleaning. Then, the seeds were sown in quartz-filled plastic containers (30 cm × 20 cm) for germination. Distilled water was poured into the containers until just over the quartz, and the containers were covered with plastic wrap to prevent the water from evaporating quickly. After germination, the distilled water was poured away. Then, the Sudan grass was watered with full-strength Hoagland’s solution. The plastic containers were kept in an illumination incubator, the temperature was 25 °C, the light intensity was 100 µmol/(m·s), and the day/night cycle was 16 h and 8 h at Sichuan Agricultural University.

### 2.2. Material Treatments

When the number of leaves reached 4–6, the materials were subjected to stress treatments. For acid aluminum stress, seedlings were cultivated with 0.1 mol/L AlCl_3_ for 5 days. For methyl jasmonate treatment, seedlings were grown with 200 µmol/L methyl jasmonate for 5 days. To induce salt stress, the seedlings underwent a 5-day cultivation period with 200 mmol/L NaCl. To induce drought stress, seedlings underwent 15% (*w*/*v*) PEG 6000 for 5 days. Meanwhile, the control group was irrigated with Hoagland’s solution. Each treatment was tested three times independently. Samples were harvested at 0, 1, 2, 3, and 5 days under the five treatments. Then, all the samples were stored at −80 °C.

### 2.3. Total RNA Extraction and First-Strand cDNA Synthesis

Total RNA was extracted from the Sudan grass material using the HiPure Plant RNA Mini Kit (Magen Biotechnology Co., Ltd., Guangzhou, China). Afterward, the purity and concentration of total RNA were measured by a spectrophotometer (Thermo Fisher Scientific, Waltham, MA, USA). Total RNA (1 µg) was reverse-transcribed using an M5 Super plus qPCR RT kit with gDNA remover (Mei5 Biotechnology Co., Ltd., Beijing, China) into cDNA. Then, the cDNA samples were stored at −20 °C.

### 2.4. Screening Candidate Reference Genes and PCR Primer Design

Through preliminary experiments, seven common reference genes (*eIF4α*, *PP2A*, *HIS3*, *UBQ9*, *UBQ10*, *Actin*, and *ACP2*) and one unigene obtained from the Sudan grass transcriptome data (*Isoform0012931*) were selected. Primer Premier version 5.00 was used to design the primers for the qRT-PCR (Table 1).

### 2.5. qRT-PCR Amplification Procedure

The qRT-PCR was carried out with a Bio-Rad CFX96 quantitative PCR instrument (BIO-RAD, Hercules, CA, USA). The 10 μL reaction mixture contained 1 μL of quintuple-diluted cDNA, 5 μL of 2× M5 HiPer SYBR Premix EsTaq (Mei5 Biotechnology Co., Ltd., Beijing, China), 0.5 µL of reverse primer and forward primer (10 µmol/L) (total 1 µL), and 3 μL of DNase/RNase-free water. The amplification procedure was 95 °C for 30 s, 40 cycles of 95 °C for 3 s, and 60 °C for 30 s. In order to confirm the specificity of the primers, the melting curve was included. The technical and biological samples were each run in triplicate for all qRT-PCRs. Each gene was able to have a standard curve established through the process of quadruple continuous dilution of the original cDNA, and 4^−1^, 4^−2^, 4^−3^, 4^−4^, 4^−5^ were used as the template. The formula for the amplification efficiency (E) was E = 10^−1/slope^.

### 2.6. Data Analysis and Validation of Selected Candidate Reference Genes

The stability of the eight reference genes was assessed through geNorm (https://seqyuan.shinyapps.io/seqyuan_prosper/, accessed on 12 October 2023), NormFinder [21], and BestKeeper [12]. Then, RefFinder (http://blooge.cn/RefFinder/?type=reference, accessed on 10 October 2023) was used to evaluate the stability comprehensively. The two top-ranked genes (*eIF4α* and *PP2A*) for drought stress and one low-ranked gene (*Actin*) were used to calculate target genes’ (*Isoform0007606* and *Isoform0002387*) expression levels to verify the reliability of the reference genes.

## 3. Results

### 3.1. Verification of Primer Specificity and Effectiveness

**Figure 1:**
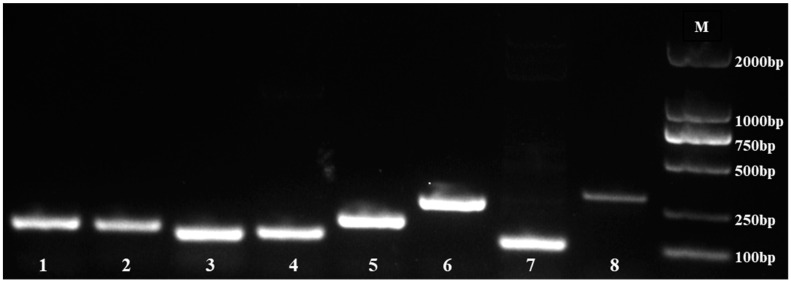
PCR products of 8 reference genes. M, DNA marker; 1, *eIF4α*; 2, *PP2A*; 3, *HIS3*; 4, *UBQ9*; 5, *UBQ10*; 6, *Isoform0012931*; 7, *ACP2*; 8, *Actin*.

We used agarose gel (1%) electrophoresis to identify the specificity of the primers. The results indicated that eight candidate reference genes (*eIF4α*, *PP2A*, *HIS3*, *UBQ9*, *UBQ10*, *Actin*, *ACP2*, and *Isoform0012931*) were amplified with a single band (Figure 1), and no primer dimer existed.

**Figure 2:**
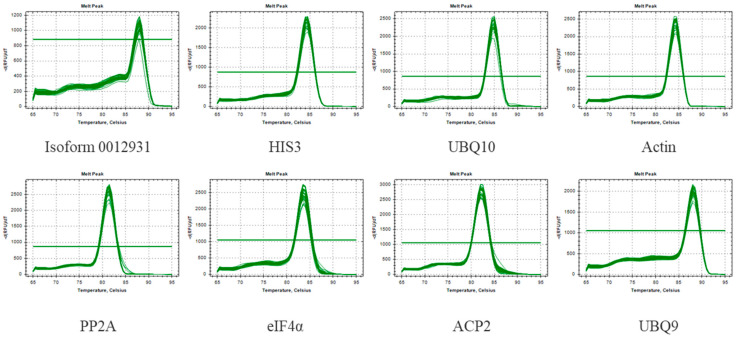
Melting curves for eight genes of CK. Melting temperatures were visualized by plotting the negative first derivative of fluorescence relative to the temperature in Celsius (–(d/dT)).

Moreover, qRT-PCR was carried out using the cDNA template of Sudan grass under different stresses, and the melting curves were analyzed. It was found that each melting curve indicated a single peak with good repeatability (Figure 2). As shown in Table 1, E ranged from 88.2% to 116.7%, and the correlation coefficient (R^2^) ranged between 0.972 and 0.996. Based on these results, it appears that the qRT-PCR primers demonstrated satisfactory specificity and efficiency, which means they were suitable for use in subsequent experiments.

### 3.2. Expression Distribution of Eight Candidate Reference Genes

**Figure 3:**
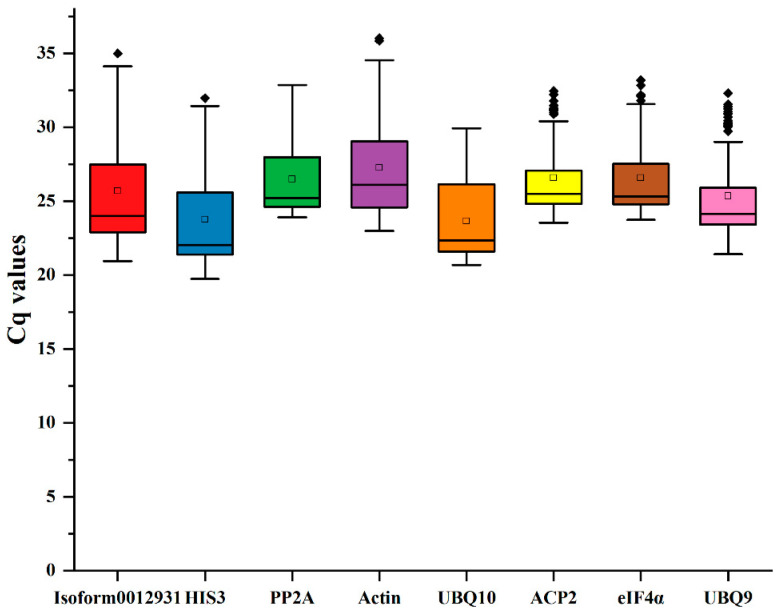
Ct values of 8 reference genes in Sudan grass under five treatments. Different colors represent different reference genes. The reference gene represented by each color corresponds to the name of the reference gene in the vertical coordinate below.

According to the findings, Ct values were used to indicate the expression levels of the reference genes, with higher gene expression levels corresponding to lower Ct values. After analyzing the Ct values we obtained from Sudan grass, it was observed that the range of Ct values was between 19.73 and 36.03. The ranking of the expression levels was *UBQ10* > *HIS3* > *UBQ9* > *Isoform0012931* > *PP2A* > *ACP2* > *eIF4α* > *Actin* (Figure 3). Among the eight reference genes, *UBQ10* had the highest mean Ct value (23.66), while *Actin* had a low expression level in comparison with the other genes (27.27).

### 3.3. Expression Stability Analysis of Candidate Reference Genes under Five Treatments

We employed four analytical tools to evaluate the eight reference genes in this research. Comprehensive rankings of the eight reference genes are listed in Table 2. We found that different reference genes exhibited different expression stability under different treatments. Meanwhile, using different analytics software can result in different rankings.

#### 3.3.1. GeNorm Analysis

**Figure 4:**
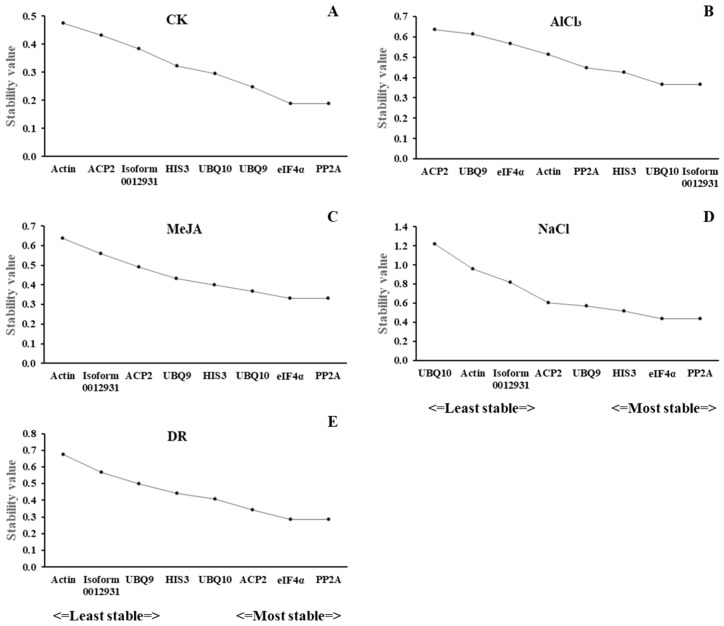
Expression stability values of 8 candidate reference genes validated by geNorm analysis. (**A**) CK, (**B**) acid aluminum stress, (**C**) methyl jasmonate treatment, (**D**) salt stress, and (**E**) drought stress.

GeNorm was created by Vandesompele et al. in 2002 to assist with screening reference genes, determining the ideal quantity of reference genes, and assessing reference genes by calculating their M value [22]. The reference gene’s stability improves as the M value decreases. In this study, *PP2A* and *eIF4α* were the optimal genes with the same M values of 0.188, 0.331, 0.438, and 0.284 in CK, MeJA, NaCl, and DR, while *Actin* was the least stable gene, with M values of 0.476, 0.639, and 0.674 in CK, MeJA, and DR. *UBQ10* had the highest M value of 1.217 in NaCl. For acid aluminum stress, *UBQ10* and *Isoform0012931* had the highest stability, whereas *ACP2* had the lowest. Out of all the treatments, *PP2A* and *eIF4α* exhibited higher stability, whereas *Actin* had the lowest (Figure 4A–E).

**Figure 5:**
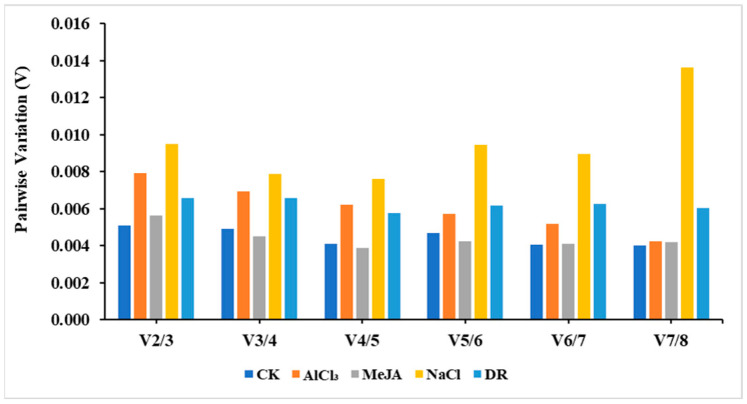
Pairwise variation (V) analysis of candidate reference genes.

Additionally, the paired variant V value of the standardized factor can be calculated by the software after introducing a new reference gene. If V_n_/V_n+1_ < 0.15, then the ideal number of reference genes would be n. On the other hand, if V_n_/V_n+1_ > 0.15, then the number would be n + 1. Based on the data in Figure 5, it appears that using just two reference genes is enough to normalize target genes across various treatments, as all pairwise variations were below 0.15.

#### 3.3.2. NormFinder Analysis

**Figure 6:**
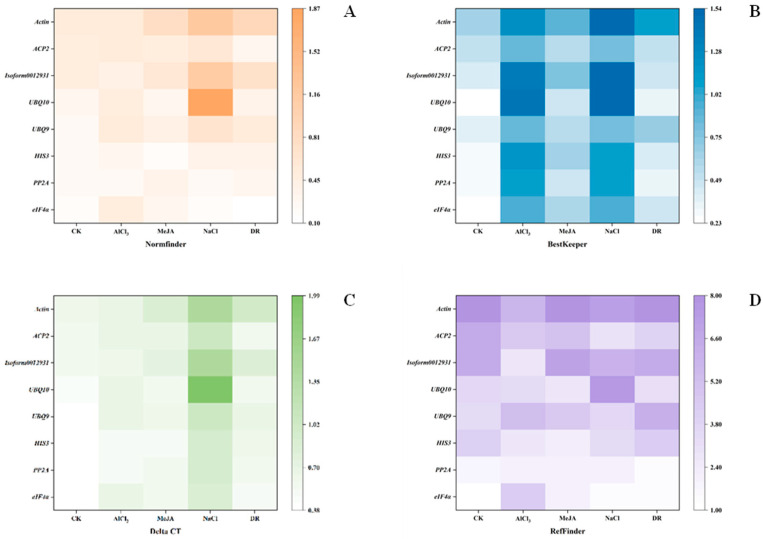
Stability analysis of 8 candidate reference genes using four methods. The color within a pane becomes whiter as the value decreases. (**A**) Analysis results from NormFinder (**B**) Analysis results from BestKeeper (**C**) Analysis results from Delta CT (**D**) Analysis results from RefFinder.

NormFinder was designed by Andersen et al. in 2004 to screen reference genes for stability [21]. It first evaluates the variance of reference genes within and between groups. Then, the reference gene with the lowest expression stability value is the most suitable [23]. The stability values of the five treatments are shown in Figure 6A. *eIF4α* was the top rank in CK, NaCl, and DR with stability values of 0.17, 0.181, and 0.098; *HIS3* was the top-ranked gene in MeJA with a value of 0.177, and *PP2A* was the top in AlCl_3_ with a value of 0.258. *Actin* had the lowest values in CK, MeJA, and DR, which is the same as geNorm.

#### 3.3.3. BestKeeper Analysis

BestKeeper is a program designed by Michael et al. to rank reference genes and target genes based on their stabilities. Through the calculations of this program, we can obtain the correlation coefficient (r), standard deviation (SD), and coefficient of variation (CV) of a pairing. A high value of r corresponds to a low SD and CV, indicating greater stability of the reference gene [12]. Figure 6B displays the findings. *eIF4α* was the top rank in CK. *PP2A* was the top rank in MeJA and DR. *ACP2* was the top rank in AlCl_3_ and NaCl. However, *Actin* was the last one in all treatments except AlCl_3_. And for AlCl_3_, *UBQ10* was the most unstable gene.

#### 3.3.4. Comparative ΔCt Analysis

To determine the expression stability differences between samples, the mean standard deviation (SD) was ranked using the ΔCt method, the stability increasing as the SD decreased [24]. *eIF4α* had the highest stability in CK, NaCl, and DR with SD of 0.38, 0.9, and 0.52. *HIS3* was the top rank in MeJA, with an SD of 0.52. *PP2A* was at the top of the ranking in AlCl_3_ with an SD of 0.54 (Figure 6C). *Actin* had the lowest values in CK, MeJA, and DR, which is the same as for geNorm and NormFinder.

#### 3.3.5. RefFinder Analysis

RefFinder is an innovative analysis software that utilizes a combination of four computational methods and calculates the geometric mean of gene weights to provide a comprehensive ranking. Stable gene expression is typically associated with a lower geometric mean [25]. In CK, NaCl, and DR, *eIF4α* was the top-ranked gene. *PP2A* was the top rank in MeJA and AlCl_3_. Nevertheless, *Actin* was the most unstable gene in four stresses, CK, MeJA, Al, and DR (Figure 6D). And for NaCl, *UBQ10* was the most unstable.

### 3.4. Validation of Candidate Reference Genes

To ensure the dependability of the rankings for the reference genes, the relative expression trend in the target genes was analyzed using the two most stable reference genes (*eIF4α*, *PP2A*), one combined reference gene (*eIF4α* + *PP2A*), and the most unstable reference gene (*Actin*) under drought stress. Two target genes (*Isoform0007606* and *Isoform0002387*) that belong to the drought-stress-response genes are highly expressed according to transcriptomic data of Sudan grass under drought stress [26]. They belong to COR (*Isoform0007606*) and MAPK (*Isoform0002387*). The polypeptide encoded by the COR gene can form a hydrophilic lipid α-helix, which can stabilize the structure of the cell membrane and make the plant resistant when the plant is dehydrated by low temperature, drought, or osmotic stress [27]. Under drought stress, cotton activates the MAPK cascade GhMAP3K62-GhMKK16-GhMPK32 pathway and phosphorylates downstream target gene GhEDT1, thereby activating the expression of ABA synthesizing gene GhNCED3, promoting ABA accumulation, and thus, participating in the molecular mechanism of drought response [28]. Therefore, they are appropriate for the target genes.

**Figure 7:**
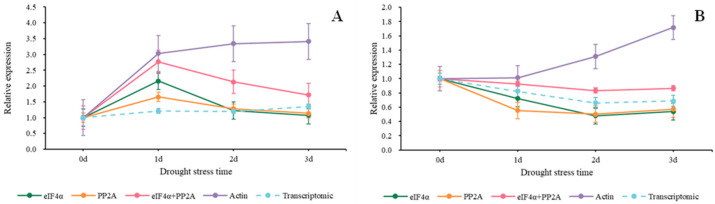
Relative expression trends of two target genes under drought stress. The target genes we used were screened from Sudan grass transcriptome data. (**A**) COR (*Isoform0007606*), (**B**) MAPK (*Isoform0002387*).

In Figure 7, we used the 2^−ΔΔCt^ method to normalize the target genes. When *Actin* served as a reference gene, we discovered that the relative expression values of the two target genes were higher than those of other groups. When *Isoform0007606* was the target gene, the relative expression values using *eIF4α*, *PP2A*, *eIF4α* + *PP2A*, and *Actin* were higher on the first day compared to the beginning. Then, the values of *eIF4α*, *PP2A,* and *eIF4α* + *PP2A* started a steady decline while the values of *Actin* started to rise (Figure 7A). As shown in Figure 7B, when using *Actin*, the expression trend of *Isoform0002387* continued to rise from the beginning, and by the third day, it was 1.7 times that of beginning Nevertheless, the values of *eIF4α*, *PP2A,* and *eIF4α* + *PP2A* started a steady decline from the beginning to the second day. There was a slight rise on the third day.

## 4. Discussion

Ideally, reference genes remain consistent across all experimental conditions and are stable in all tissues and growth stages, but it has been shown in many studies that stable expression genes will change across diverse species and conditions [14,29]. An effective way to analyze gene expression is through the use of qRT-PCR, known for its high efficiency. However, choosing appropriate reference genes is crucial when analyzing gene expression levels, as the accuracy of the measurements will be impacted by the RNA quality, efficiency of reverse transcription, or cDNA quality [30].

Sudan grass is an inexpensive, fast-growing plant that has great forage value, but there are not studies on reference gene screening of Sudan grass under different stresses [9]. Therefore, we screened eight reference genes from transcriptome data and common reference genes using five algorithms. As can be seen from Table 2, most of the rankings were different between the five algorithms. However, comparative ΔCt and NormFinder had the same order for CK, methyl jasmonate treatment, and salt stress. Meanwhile, comparative ΔCt and RefFinder had the same ranking for DR. Under aluminum stress, *UBQ9* ranked second in BestKeeper, but in the other four algorithms it ranks very low. In comparative ΔCt, NormFinder, and RefFinder, *HIS3* was the top rank in MeJA, but it was an unstable reference gene in BestKeeper. So, the ranking of BestKeeper is always significantly different from those of the other four algorithms.

Due to drought and the application of large amounts of fertilizer or other factors, global soil salinization is becoming increasingly serious, and there are about 10 million km^2^ of saline–alkali land in the world. Therefore, more and more attention has been paid to the research of saline soils [31]. It can be seen from the result of the ranking that *eIF4α* and *PP2A* are the most stable under CK, MeJA, NaCl, and DR *eIF4α* belongs to the eukaryotic initiation factor 4α family. It is required for the binding of mRNA to 40S ribosomal subunits. It can also unwind double-stranded RNA [32]. *PP2A* belongs to the phosphoprotein phosphatase family of non-metal dependent serine/threonine protein phosphatases. They are universal in eukaryotes. *PP2A* regulates plant vesicle traffic and various processes of plant development and stress responses [33]. The results are consistent with the sequencing results of reference genes of sorghum. For example, Ma et al. found that *GAPDH* and *eIF4α* had the highest expression stability in leaves of sorghum under low nitrogen treatment, while *Actin* had the lowest [34]. Palakolanu et al. found that *PP2A* and *CYP* were the reference genes with the best performance under abiotic stress in sorghum [35]. María et al. found that *ARI 8* and *PP2A* were the top ranks under glyphosate stress in sorghum [36]. Otherwise, *eIF4α* and *PP2A* always express stably in *Larix olgensis*, *Vaccinium corymbosum* × *angustifolium*, *Chenopodium quinoa* Willd, oat, *Suaeda glauca* L., and so on, especially in salt stress and drought stress [37,38,39,40,41].

However, in our study, *Actin* is the most unstable gene in most treatments. This result is similar to the research of Zhang et al. in *Papaver somniferum* L. but contrary to the study of You et al. in *Prunus persica* [42,43]. Studies on the reference genes in various plants at different growth stages showed that *Actin* and *UBQ* were the most stable [44]. But our study researched different abiotic stresses, which is probably the reason why *Actin* and *UBQ* express unstably. Additionally, the reference gene we screened from the transcriptome data (*Isoform0012931*) usually had a poor performance, always being ranked sixth or seventh. Nevertheless, its performance was better in aluminum stress and it could be ranked in the top three.

In conclusion, we identified eight reference genes in Sudan grass under four abiotic stresses and used five arithmetics for evaluation. Because of the use of different algorithms, the ranking results are also different. The best reference gene was *PP2A* under all treatments. *eIF4α* can be used as a reference gene in CK, MeJA, NaCl, and DR. *HIS3* can be used as an optimal reference gene in AlCl_3_. *Actin* was the most unstable in most treatments. Our research can provide data support for the next step of functional analysis of Sudan grass. However, it still has room for improvement; we will keep investigating more reference genes in various tissues, under various stresses, and in various varieties of Sudan grass.

## Figures and Tables

**Table 1:** Primer sequences (forward and reverse 5′-3′) of eight candidate reference genes and two target genes.

Gene Symbol	Gene Description	Forward/Reverse Primer (5′-3′)	Primers Tm (°C)	Amplicon Length (bp)	RT-qPCR Efficiency (%)	R^2^
eIF4α	Eukaryotic initiation factor 4 α	CAACTTTGTCACCCGCGATGA TCCAGAAACCTTAGCAGCCCA	57.57 57.57	178	99.5	0.993
PP2A	Protein phosphatase 2A	AACCCGCAAAACCCCAGACTA TACAGGTCGGGCTCATGGAAC	57.57 59.52	176	111.3	0.980
HIS3	Histone H3	ACTTCAAGACTGATCTGCGTTTCC CATGGATGGCACAAAGGTTGG	57.86 57.57	146	102.2	0.988
UBQ9	Polyubiquitin 9	TACAGTTCTACAAGGTGGACGAC GCAGTAGTGGCGGTCGAAGT	57.77 59.50	158	92.7	0.992
UBQ10	Polyubiquitin 10	CCGTGGTGGCCAGTAAGTTC GGACTCAACATGGGCTCTGC	59.50 59.50	204	88.2	0.987
Isoform0012931		ATGGCCAACCGCTGGGTC CTTGGGGTCAGTGAAGAACTTGT	59.46 57.77	286	116.7	0.972
ACP2	Acyl carrier protein 2	ACGAACTTGTTGCGGCAGAAG GAACAAGAAGGGATGCGCTGG	57.57 59.52	128	116.3	0.988
Actin	Actin gene	AAGTGCGACGTGGATATTAGGA TCTTGGGCGGAAAGAATTAGA	55.81 53.66	344	101.5	0.996
Isoform0007606	Cold response gene	TTCGGCACTTCCTTCCTCA GAATAGACGCAGAATAACAGCAATA	58.70 58.40	204		
Isoform0002387	Mitogen-activated protein kinase	CCGAGCAATTTGTTCCTAA GCATCATCTGGTGAGCCTAT	53.80 54.70	284		

**Table 2:** Comprehensive rankings of 8 candidate reference genes.

Rank	1	2	3	4	5	6	7	8
(A) CK								
Delta CT	*eIF4α*	*PP2A*	*UBQ9*	*HIS3*	*UBQ10*	*ACP2*	*Isoform0012931*	*Actin*
BestKeeper	*eIF4α*	*UBQ10*	*PP2A*	*HIS3*	*UBQ9*	*Isoform0012931*	*ACP2*	*Actin*
Normfinder	*eIF4α*	*PP2A*	*UBQ9*	*HIS3*	*UBQ10*	*ACP2*	*Isoform0012931*	*Actin*
GeNorm	*PP2A*|*eIF4α*		*UBQ9*	*UBQ10*	*HIS3*	*Isoform0012931*	*ACP2*	*Actin*
RefFinder	*eIF4α*	*PP2A*	*UBQ9*	*UBQ10*	*HIS3*	*ACP2*	*Isoform0012931*	*Actin*
(B) Salt stress								
Delta CT	*eIF4α*	*PP2A*	*HIS3*	*ACP2*	*UBQ9*	*Isoform0012931*	*Actin*	*UBQ10*
BestKeeper	*ACP2*	*UBQ9*	*eIF4α*	*PP2A*	*HIS3*	*Isoform0012931*	*UBQ10*	*Actin*
Normfinder	*eIF4α*	*PP2A*	*HIS3*	*ACP2*	*UBQ9*	*Isoform0012931*	*Actin*	*UBQ10*
GeNorm	*PP2A*|*eIF4α*		*HIS3*	*UBQ9*	*ACP2*	*Isoform0012931*	*Actin*	*UBQ10*
RefFinder	*eIF4α*	*PP2A*	*ACP2*	*HIS3*	*UBQ9*	*Isoform0012931*	*Actin*	*UBQ10*
(C) Drought stress								
Delta CT	*eIF4α*	*PP2A*	*UBQ10*	*ACP2*	*HIS3*	*UBQ9*	*Isoform0012931*	*Actin*
BestKeeper	*PP2A*	*UBQ10*	*HIS3*	*eIF4α*	*Isoform0012931*	*ACP2*	*UBQ9*	*Actin*
Normfinder	*eIF4α*	*PP2A*	*ACP2*	*UBQ10*	*HIS3*	*UBQ9*	*Isoform0012931*	*Actin*
GeNorm	*PP2A*|*eIF4α*		*ACP2*	*UBQ10*	*HIS3*	*UBQ9*	*Isoform0012931*	*Actin*
RefFinder	*eIF4α*	*PP2A*	*UBQ10*	*ACP2*	*HIS3*	*UBQ9*	*Isoform0012931*	*Actin*
(D) Aluminum stress								
Delta CT	*PP2A*	*HIS3*	*Isoform0012931*	*UBQ10*	*eIF4α*	*Actin*	*UBQ9*	*ACP2*
BestKeeper	*ACP2*	*UBQ9*	*eIF4α*	*PP2A*	*HIS3*	*Actin*	*Isoform0012931*	*UBQ10*
Normfinder	*PP2A*	*HIS3*	*Isoform0012931*	*eIF4α*	*UBQ10*	*Actin*	*ACP2*	*UBQ9*
GeNorm	*Isoform0012931*|*UBQ10*		*HIS3*	*PP2A*	*Actin*	*eIF4α*	*UBQ9*	*ACP2*
RefFinder	*PP2A*	*HIS3*	*Isoform0012931*	*UBQ10*	*eIF4α*	*ACP2*	*UBQ9*	*Actin*
(E) Methyl jasmonate treatment								
Delta CT	*HIS3*	*eIF4α*	*UBQ10*	*PP2A*	*UBQ9*	*ACP2*	*Isoform0012931*	*Actin*
BestKeeper	*PP2A*	*UBQ10*	*ACP2*	*UBQ9*	*eIF4α*	*HIS3*	*Isoform0012931*	*Actin*
Normfinder	*HIS3*	*eIF4α*	*UBQ10*	*PP2A*	*UBQ9*	*ACP2*	*Isoform0012931*	*Actin*
GeNorm	*PP2A*|*eIF4α*		*UBQ10*	*HIS3*	*UBQ9*	*ACP2*	*Isoform0012931*	*Actin*
RefFinder	*PP2A*	*eIF4α*	*HIS3*	*UBQ10*	*UBQ9*	*ACP2*	*Isoform0012931*	*Actin*

## Data Availability

Data are contained within the article.
